# Effect of statins and antihyperglycemics on chronic kidney disease in patients with type 2 diabetes mellitus: a retrospective cohort study with a 12-year follow-up

**DOI:** 10.1080/20523211.2024.2414293

**Published:** 2024-12-20

**Authors:** Ammar Abdulrahman Jairoun, Chong Chee Ping, Baharudin Ibrahim, Dina Farhan Al Jawamis, Asma Khaled Al Jaberi, Tasnim Dawoud, Khuloud Jamal Mohammed, Faris El-Dahiyat, Moyad Shahwan

**Affiliations:** a Discipline of clinical pharmacy, School of Pharmaceutical Sciences, Universiti Sains Malaysia (USM), Pulau Pinang, Malaysia; b Faculty of Pharmacy, Universiti Malaya, Kuala Lumpur, Malaysia; c Pharmacy Department, Tawam Hospital, SEHA, Al Ain, United Arab Emirates; d Endocrinology Department, SEHA, Al Ain, United Arab Emirates; e Clinical Pharmacy Program, College of Pharmacy, Al Ain University, Al Ain, United Arab Emirates; f AAU Health and Biomedical Research Center, Al Ain University, Abu Dhabi, United Arab Emirates; g College of Pharmacy and Health Sciences, Ajman University, Ajman, UAE.; h Centre of Medical and Bio allied Health Sciences Research, Ajman University, Ajman, United Arab Emirates

**Keywords:** Statin, Type 2 diabetes mellitus, cohort study, metformin, diabetic nephropathy, chronic kidney disease

## Abstract

**Background:**

Chronic Kidney Disease (CKD) represents a significant worldwide health challenge, with far-reaching implications for both patients and healthcare systems. This study aimed to identify the incidence of CKD at stages 3–5, analyzed the impact of statin and other antihyperglycemic interventions, on the CKD progression in individuals with T2DM.

**Methods:**

This was a single-center retrospective cohort study based on data derived from electronic medical records (EMR) of UAE populations with diabetes mellitus, registered at outpatient clinics at Tawam Hospital in Al Ain, UAE, between January 2011 and December 2021. T2DM patients aged ≥ 18 years who had serum HbA1c level ≥ 6.5% and using one of the statin therapies were inclusion criteria. Patients with T1DM, who had undergone permanent renal replacement therapy, with under 1 year of follow-up and missing or incomplete data were excluded from the study. The collected data encompassed socio-demographics, detailed medical history, anthropometric measurements, laboratory analyses, clinical parameters, disease characteristics, and medications.

**Results:**

Our study included a cohort of 1,003 individuals. We observed 388 subjects developed CKD stages 3–5 across an average monitoring duration of 11.7 years. This resulted in a cumulative incidence of 38.7%, translating to an incidence rate of 38 cases per 1000 person-years. There was a statistically significant difference in the cumulative incidence of CKD stages 3 ± 5 according to statin therapy (*P* = 0.047). High intensity statin users are more likely to develop a CKD stage 3–5 compared to low/moderate intensity users and to no statin users respectively (44.3% vs 37.9%), (44.3% vs 30.9%). Conversely, the use of Biguanides was associated with a decreased probability of CKD progression (37.9% vs. 52.8%; *P* = 0.001), whereas Insulin users demonstrated a heightened risk (54.2% vs. 34.1%; *P* < 0.001).

**Conclusion:**

The findings emphasise the pivotal role of personalised treatment strategies, particularly concerning statin therapy and other medications, in populations at high risk.

## Background

The International Diabetes Federation (Shi & Hu, 2014) predicts a significant rise in global diabetes cases, projecting an increase from 382 million in 2013 to an estimated 592 million by 2035. Within the realm of diabetes-related complications, diabetic nephropathy (DN) emerges as a predominant cause of end-stage renal disease, posing a substantial and perilous chronic health threat (Guo et al., 2015). However, options for halting the progression of diabetic nephropathy remain limited, primarily revolving around angiotensin II-receptor blockers (ARB) and angiotensin-converting enzyme inhibitors (ACEI). This necessitates the exploration of alternative approaches to preserve kidney function.

Numerous plausible pathways leading to renal impairment in diabetic nephropathy have been uncovered. Hyperlipidemia emerges as a significant contributor to the development of diabetic kidney disease (DN), potentially inflicting harm on mesangial cells through lipotoxicity or by promoting intrarenal atherosclerosis (Fried et al., 2001; Guijaro & Keane, 1996; Tonelli et al., 2003).

The cornerstone in managing dyslipidemia is the use of HMG-CoA reductase inhibitors, commonly known as statins, which have demonstrated efficacy in reducing the risk of cardiovascular morbidity and mortality (Koo, 2014). Additionally, emerging from fundamental research, statins may offer renal protection through anti-inflammatory and anti-proliferative mechanisms (Campese & Park, 2007). However, the precise impact of statins on renal function in clinical settings remains a subject of debate. The degree of hypercholesterolemia appears to correlate with proteinuria, with experimental studies revealing that dyslipidemia exacerbates glomerular and interstitial damage (Keane, 2000). Beyond their cholesterol-lowering effects, statins exert influence on critical intracellular pathways tied to inflammatory and fibrogenic responses, constituting the primary route leading to progressive renal impairment (Abrass, 2004; Oda & Keane, 1999). Furthermore, statins are recommended for use in patients with chronic kidney disease (CKD) due to their positive effects on endothelial function, suppression of monocyte recruitment, attenuation of mesangial matrix accumulation and mesangial cell proliferation, antifibrotic properties, as well as antioxidative and anti-inflammatory cytokine effects (Campese et al., 2005; Keane, 2000).

Despite disagreements regarding the impact of statin therapy on kidney function in humans, a growing body of evidence suggests that statins possess renoprotective potential (Jandeleit-Dahm et al., 1999; Molnar et al., 2011; Welten et al., 2008). Large population-based retrospective cohort research indicates that preoperative statin use is associated with a reduced incidence of acute renal injury and the need for acute dialysis following major elective surgeries (Molnar et al., 2011). Additionally, statin use is correlated with accelerated recovery from kidney injury and a decreased risk of all-cause mortality following vascular surgeries (Welten et al., 2008). A meta-analysis research also observed positive alterations in GFR, albuminuria, and proteinuria among statin-treated individuals (Sandhu et al., 2006).

Conversely, another systematic review and meta-analysis encompassing 143,888 adult patients found that while statins slightly reduced proteinuria and decelerated the decline in glomerular filtration rate in adult non-dialysis patients, they did not prevent acute kidney injury (AKI) (Su et al., 2016). In patients with CKD not requiring dialysis and without baseline cardiovascular disease, a separate Cochrane analysis consistently demonstrated that statin therapy reduced mortality and prevented severe cardiovascular events (Rodrigues, 2015). However, for patients undergoing hemodialysis, statin medication did not yield the same benefits as indicated by numerous recent meta-analyses (Hou et al., 2013; Navaneethan et al., 2009; Palmer et al., 2012; Strippoli et al., 2008; Upadhyay et al., 2012; Zhang et al., 2014). Nevertheless, none of these trials have investigated the impact of high-intensity statin therapy on clinical outcomes in CKD patients. Additionally, the 2013 KDIGO (kidney disease: Improving Global Outcomes) clinical practice guideline does not specify statin dosages despite recommending statin initiation in CKD patients (Tonelli & Wanner, 2014). The potential advantages of high-intensity statin therapy in this specific population remain uncertain. Furthermore, heightened clinical practice carries elevated risks of harm and complications, necessitating a thorough examination. Consequently, to explore the incidence of CKD stages 3 ± 5 in type 2 diabetic individuals, we conducted this retrospective cohort study encompassing high-intensity statin medication, moderate/mild-intensity statin therapy, and a placebo group.

## Methods

### Research design

This is Single center retrospective cohort study based on data that obtained from electronic medical records (EMR) of UAE populations with diabetes mellitus, registered at outpatient clinics at Tawam Hospital in Al Ain, UAE, between January 2011 and December 2021. Notably, the hospital’s clinical laboratory has obtained ISO-9002 certification, making it the first Middle Eastern medical facility to achieve this distinction. This certification has been recognised by thirty countries, affirming the laboratory’s commitment to quality control.

### Study population

The study population is the type 2 diabetes mellitus patients registered at outpatient clinics at Tawam Hospital in Al Ain, UAE.

### Subject criteria (inclusion/exclusion criteria)

The study’s inclusion criteria encompassed Type 2 diabetes mellitus patients aged 18 years or older with a serum HbA1c level equal to or greater than 6.5%, diagnosed by a physician or receiving diabetes medications. Additionally, participants were required to be using one of the specified statin therapies. Exclusion criteria involved patients with chronic kidney disease (CKD) who had undergone permanent renal replacement therapy, those with type 1 diabetes mellitus, individuals with less than one year of follow-up or missing/incomplete data, as well as patients with COVID-19, cancer, or HIV/AIDS.

### Sample size estimation

We determined the sample size for our cohort study using OpenEpi software and the following cohort study sample size formula: https://www.openepi.com/SampleSize/SSCohort.htm. The sample size was determined in line with the study’s primary objective – to assess the impact of statin therapy on the development of chronic kidney disease (CKD) in patients with type 2 diabetes mellitus in the United Arab Emirates. We employed a two-sided test with a power of (1−β) = 0.95 and a significance level of α = 0.05. The control-to-case ratio was set at one. Based on an anticipated risk ratio of 1.48 (Chung et al., 2013) and an assumed proportion of controls with outcome = 6, our calculations indicated a minimum required sample size of 2580 participants.

### Sampling method and subject recruitment

Purposeful sampling was used in the recruitment of patients. The patients recruited retrospectively from the electronic medical records (EMR) of UAE populations with type 2 diabetes mellitus, registered at outpatient clinics at Tawam Hospital in Al Ain, UAE.

### Research tool

A data collection form (in excel sheet) was designed for the data collection purposes. The data collected for each case subject included subject ID number and baseline covariates of the patients. The patients’ characteristics was collected at baseline. The collected data included: (i) Socio-demographics, (ii) detailed medical history, (iii) anthropometric measurements, (iv) laboratory analyses and clinical parameters, (v) disease characteristics and medications.

### Operational definition

- **Diagnosis of diabetes mellitus (DM):** American Diabetes Association criteria of HbA1c ≥ 6.5%will be used to define DM. Diagnosis established by a physician.
- **CKD stages 3 ± 5:** were defined by using the KDIGO 2012 Clinical Practice Guideline for the Evaluation and Management of Chronic Kidney Disease was used in the study as an eGFR < 60 mL/min/1.73 m^2^ for ≥ 3 months (CKD, 2021).

### Data collection method

This is a retrospective cohort study based on data obtained from electronic medical records (EMR) of UAE populations with type 2 diabetes mellitus, registered at outpatient clinics at Tawam Hospital in Al Ain, UAE, between January 2011 and December 2021. The annual follow up data for this retrospective cohort study were collected from January 2011 to December 2021 to establish the risk factors surveillance of the CKD in type 2 diabetes mellitus.

**Figure 1.**
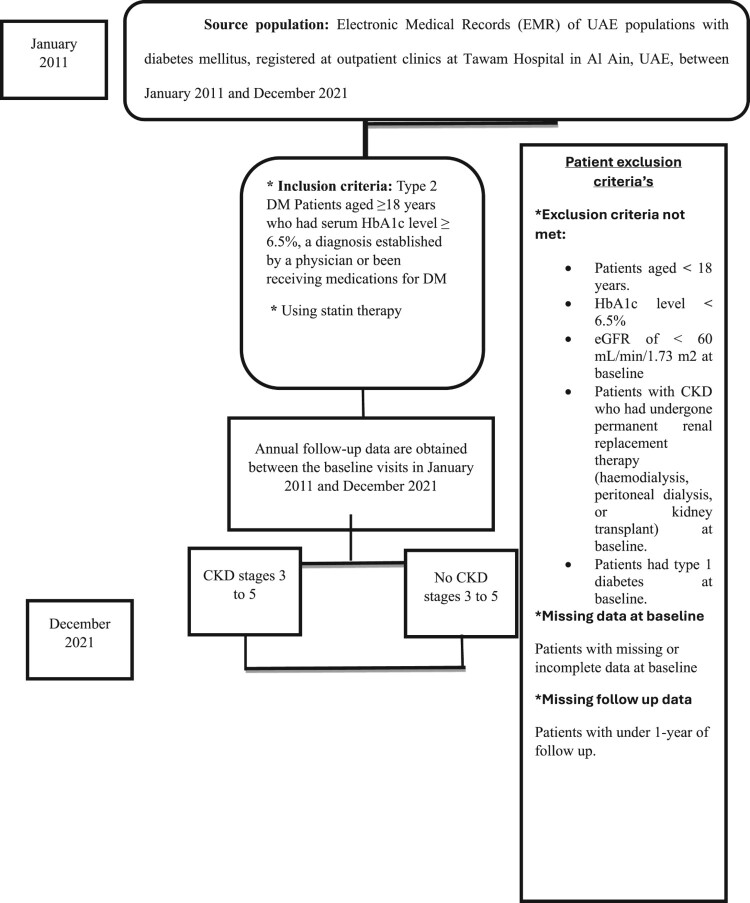
Flow chart of study.

The baseline clinical variables, demographic data and time to event (development of CKD stages 3 to stage 5) was collected and reported from electronic data base system. We identified the diabetic patients with a unique reference number, which will ensue that the patient met the inclusion criteria, and this in turn reduce the reporting error. The estimated glomerular filtration rate (eGFR) was repeatedly evaluated for each patient every three months, starting from baseline to December 2021. Also, the follow-up laboratory tests were revised and monitored to ensure that patients fitful the inclusion criteria and this will enhance the quality of the collected data. The sampling methods was convenient sampling based on the inclusion and exclusion criteria (Figure 1).

### Statistical analysis

The Statistical Package for Social Science (SPSS) Version 26 software was employed for data entry, coding, and analysis. Baseline characteristics of patients who did and did not develop CKD stages 3–5 were compared using independent samples t-tests and one-way ANOVA. For non-normally distributed continuous variables, the Mann–Whitney U test and Kruskal–Wallis test were utilised, while categorical variables were assessed using χ2 or Fisher’s exact test (two-tailed). Missing covariate and dependent variable data were excluded from the analysis, with no data imputation applied. The period of observation or Years-Patient at Risk of Developing Stages of CKD 3 ± 5 was computed for each patient from their 2011 baseline visit until their last outpatient appointment or the diagnosis of CKD stages 3 ± 5. The incidence rate was calculated by dividing the number of new cases of CKD stage 3 ± 5 by the patient-years at risk. Survival time was determined and compared across different patient groups using the Kaplan–Meier method and the log-rank test. Statistical significance was determined using *p*-values less than 0.05.

## Results

### Patient enrolment process

**Figure 2.**
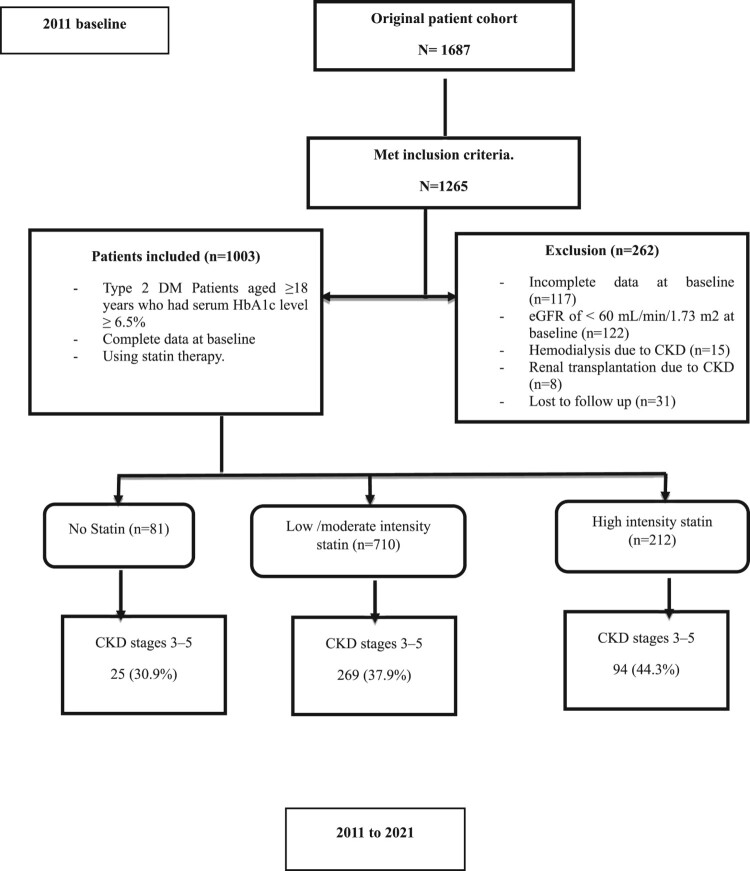
Flow diagram of the patient cohort.

Out of the 1265 patients who met the criteria, 262 patients were found to be ineligible for various reasons. Among them 122 had eGFR levels below 60 mL/min/1.73 m^2^, 15 required hemodialysis due to CKD, 8 had undergone transplantation and 86 had data incomplete baseline HbA1c, Urea, Albumin, TC/HDL C ratio and Vitamin D levels. Moving forward from the baseline until December 26th, 2021 we monitored the eGFR every three months for each patient. Unfortunately during this period we couldn’t obtain follow up measurements for Urea and SCr from 31 patients which led to their exclusion due to loss of follow up. As a result of these considerations a total of 1003 patients with an eGFR to or greater, than 60 mL/min/1.73 m^2^ were ultimately included in this study (Figure 2).

### Demographics and baseline characteristics

Table 1 presents the demographic and comorbidities characteristics of the study patients. A total of 1003 patients were recruited in the study. The mean age of study cohort at baseline was 70.6 ± 28.2 years. Of the total, 60% (*n* = 602) were female and 40% (*n* = 401) were male. of the total subjects, 8.2% (*n* = 82) were smokers and 36.9% (*n* = 370) had DM family history. The majority of the study cohort had a history of hypertension (848/1003; 84.5%), 90.6% (*n* = 909) had a history of Dyslipidemia and 14.4% (*n* = 144) had a history of ischemic heart disease.

**Table 1.** Demographics and comorbidities of the study cohort (*n* = 1003).

	Group	Descriptive Statistics
Age (years), Mean (SD)	70.6 (28.2)	
gender *n* (%)	Female	602 (60)
	Male	401 (40)
Smoking *n* (%)	Yes	82 (8.2)
	No	921 (91.8)
DM family history *n* (%)	Yes	370 (36.9)
	No	633 (63.1)
Hypertension *n* (%)	Yes	848 (84.5)
	No	155 (15.5)
Dyslipidemia *n* (%)	Yes	909 (90.6)
	No	94 (9.4)
Ischemic heart disease *n* (%)	Yes	144 (14.4)
	No	859 (85.6)

**Abbreviation:** DM; diabetes mellitus.

Patients with CKD stages 3–5 event were older at baseline; less frequently had a DM family history and history less frequently had of hypertension but more frequently had a history of Ischemic heart disease (Table 2).

**Table 2.** Comparison of demographics and comorbidities according to CKD stages 3–5.

	Total (*N* = 1003)	CKD (*n* = 388)	No CKD (*n* = 615)	*P value
Age (years), Mean (SD)	70.6 (28.2)	76.5 (11.1)	66.8 (34.4)	< 0.001^a^
Female gender n (%)	602 (60)	222 (36.9%)	380 (63.1)	0.150
Smoking n (%)	82 (8.2)	30 (36.6)	52 (63.4)	0.684
DM family history n (%)	370 (36.9)	116 (31.4)	254 (68.6)	< 0.001
Hypertension n (%)	848 (84.5%)	371 (43.8)	477 (56.3%)	< 0.001
Dyslipidemia n (%)	909 (90.6%)	356 (39.2)	553 (60.8)	0.332
Ischemic heart disease *n* (%)	144 (14.4)	94 (65.3)	50 (34.7)	< 0.001

**Abbreviations:** DM; diabetes mellitus. CKD; eGFR < 60 mL/min/1.73 m^2^ for ≥ 3 months. No CKD; eGFR > 60 mL/min/1.73 m^2^ for ≥ 3 months.

*P-values obtained from the Chi-square (two-tailed) for categorical variables and ^a^Independent samples t-test for continuous variables.

Table 3 presents the concurrent Medication of the study cohort. Of the total study cohort, 81 (8.1%) were non statin users, 710 (70.8%) were Low to moderate intensity statin users and 212 (21.1%) were high intensity statin users.

**Table 3.** Concurrent medication of the study cohort (*n* = 1003).

Medications	Groups	Frequency	Percentage
Statin n (%)	No statin	81	8.1
	Low /moderate intensity statin	710	70.8
	High intensity statin	212	21.1
Diuretics n (%)	Yes	332	33.1
	No	671	66.9
ACEIs n (%)	Yes	263	26.2
	No	740	73.8
ARBs n (%)	Yes	509	50.7
	No	494	49.3
Alpha-blockers	Yes	83	8.3
	No	920	91.7
Beta-blockers	Yes	300	29.9
	No	703	70.1
Calcium channel blocker	Yes	301	30.0
	No	702	70.0
Sulfonylureas	Yes	545	54.3
	No	458	45.7
Thiazolidinedione	Yes	164	16.4
	No	839	83.6
Dipeptidyl Peptidase	Yes	422	42.1
	No	581	57.9
Biguanide	Yes	950	94.7
	No	53	5.3
Alpha glucosidase	Yes	39	3.9
	No	964	96.1
Insulin	Yes	227	22.6
	No	776	77.4

**Abbreviations:** ACEIs; Angiotensin-Converting Enzyme Inhibitors. ARBs; Angiotensin receptor blockers.

The antihyperglycemic medications cardiovascular medications among study cohort were as follows: 545 (54.3%) used Sulfonylureas, 164 (16.4%) used Thiazolidinedione, 422 (42.1%) used Dipeptidyl Peptidase, 950 (94.7%) used Biguanide, 39 (3.9%) used Alpha glucosidase and 227 (22.6%) used Insulin.

Table 4 illustrates the patients’ medication usage based on the development of CKD stages 3–5. Patients with CKD stages 3–5 were more frequent users of Insulin, but less frequently Biguanides.

**Table 4.** Comparison of medications according to CKD stages 3–5.

	Total (*N* = 1003)	CKD (*n* = 388)	No CKD (*n* = 615)	*P value
Statin n (%)				0.077
No statin	81 (8.1)	25 (30.9)	56 (69.1)	
Low /moderate intensity statin	710 (70.8)	269 (37.9)	441 (62.1)	
High intensity statin	212 (21.1)	94 (44.3)	118 (55.7)	
Sulfonylureas n (%)				
Yes	545 (54.3)	223 (40.9)	322 (59.1)	0.113
No	458 (45.7)	165 (36)	293 (64)	
Thiazolidinedione n (%)				
Yes	164 (16.4)	74 (45.1)	90 (54.9)	0.064
No	839 (83.6)	314 (37.4)	525 (62.6)	
Dipeptidyl Peptidase n (%)				
Yes	422 (42.1)	167 (39.6)	255 (60.4)	0.622
No	581 (57.9)	221 (38)	360 (62)	
Biguanide n (%)				
Yes	950 (94.7)	360 (37.9)	590 (62.1)	0.030
No	53 (5.3)	28 (52.8)	25 (47.2)	
Alpha glucosidase n (%)				
Yes	39 (3.9)	18 (46.2)	21 (53.8)	0.329
No	964 (96.1)	370 (38.4)	594 (61.6)	
Insulin n (%)				
Yes	227 (22.6)	123 (54.2)	104 (45.8)	< 0.001
No	776 (77.4)	265 (34.1)	511 (65.9)	

**Abbreviations:** ACEIs; Angiotensin-Converting Enzyme Inhibitors. ARBs; Angiotensin receptor blockers. CKD. eGFR < 60 mL/min/1.73 m^2^ for ≥ 3 months. No CKD; eGFR > 60 mL/min/1.73 m^2^ for ≥ 3 months.

*P-values obtained from the Chi-square (two-tailed) for categorical variables.

### Estimation of the cumulative incidence and incidence rate of CKD stages 3–5 among diabetic patients according to statin therapy

In our study cohort of the total 1003, 388 patients had newly developed CKD stages 3–5 over mean follow-up of 11.7 years (95% CI 11.4, 11.9 years). The cumulative incidence of CKD stages 3 ± 5 during this period was 38.7% (95% CI 35.7, 41.7). The incidence rate of CKD stages 3 ± 5 was 38 (95% CI 34.4, 41.6) cases per 1000 individuals-years.

**Figure 3.**
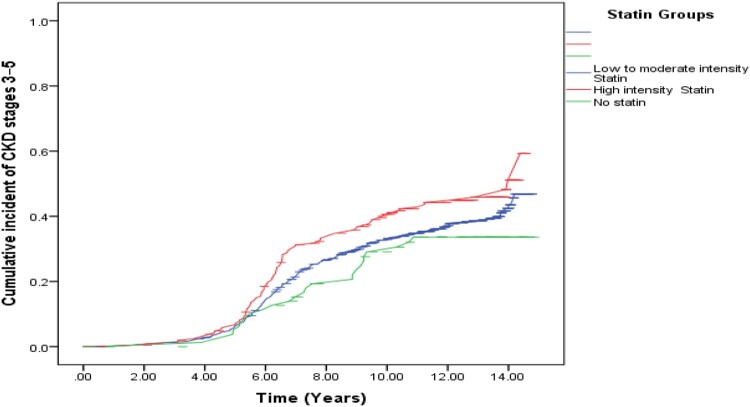
Cumulative incident of CKD stages 3–5 among statins user groups.

The results of the log-rank test showed a statistically significant difference in the cumulative incidence of CKD stages 3 ± 5 according to statin therapy (*P* = 0.047). High intensity statin users more likely to develop a CKD stages 3–5 compared to low/moderate intensity users and to no statin users respectively (44.3% vs 37.9%), (44.3%% vs 30.9%). The incidence rate of CKD stages 3 ± 5 among high intensity statin users was 45.7 (95% CI 37, 54.4) cases per 1000 individuals-years (Table 5 and Figure 3).

**Table 5.** Cumulative incident and incidence rate of CKD stages 3–5 among statins user groups.

Statin groups	Total *n* (%)	CKD *n* (%)	No CKD *n* (%)	CKD incident rate (95% CI) per 1000 person days	Log-rank test, *P*-value
**Statin intensity**					0.047
No statin	81 (8.1)	25 (30.9)	56 (69.1)	29.4 (18.2, 40.6)	
Low to moderate intensity	710 (70.8)	269 (37.9)	441 (62.1)	36.9 (32.7, 41)	
High intensity	212 (21.1)	94 (44.3)	118 (55.7)	45.7 (37, 54.4)	
Total	1003	388 (38.7)	615 (61.3)	38 (34.4, 41.6)	
**Statin medications**					
No statin	81 (8.1)	25 (30.9)	56 (69.1)	29.4 (18.2, 40.6)	0.201
Simvastatin	96 (9.6)	36 (37.5)	60 (62.5)	35.63 (24.5, 46.7)	
Rosuvastatin	182 (18.1)	59 (32.4)	123 (67.6)	31.9 (24, 39.9)	
Pravastatin	27 (2.7)	11 (40.7)	16 (59.3)	37.7 (15.5, 59.8)	
Atorvastatin	617 (61.5)	257 (41.7)	360 (58.3)	41.4 (36.7, 46.2)	
Total	1003	388 (38.7)	615 (61.3)	38 (34.4, 41.6)	

**Abbreviations:** CKD; eGFR < 60 mL/min/1.73 m^2^ for ≥ 3 months. No CKD; eGFR > 60 mL/min/1.73 m^2^ for ≥ 3 months.

There was no statistically significant difference in the cumulative incidence of CKD stages 3–5 concerning the statin medications (*P* = 0.201).

### Cumulative incidence and incidence rate of CKD stages 3–5 among diabetic patients according to antihyperglycemic medications

**Figure 4.**
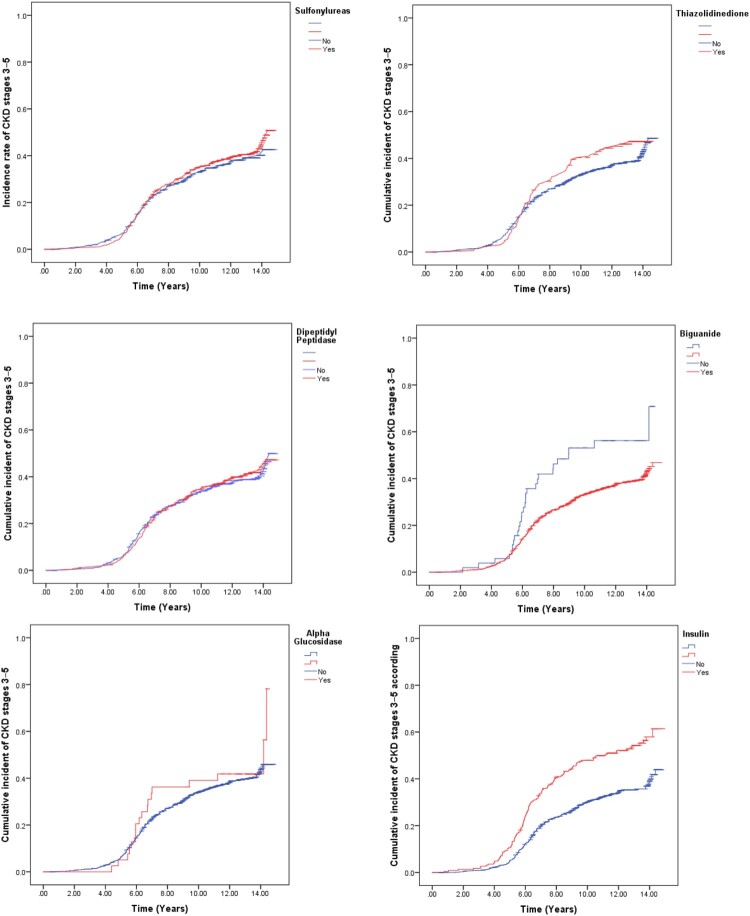
Cumulative incident of CKD stages 3–5 according to antihyperglycemic drugs.

A statistically significant difference was observed in the cumulative incidence of CKD stages 3–5 concerning the use of Biguanide and Insulin therapies. Patients using Biguanides were less likely to develop CKD stages 3–5 (37.9% vs. 52.8%; *P* = 0.001). However, patients using Insulin were more likely to develop CKD stages 3–5 (54.2% vs. 34.1%; *P* < 0.001) (Table 6 and Figure 4).

**Table 6.** Cumulative incident and incidence rate of CKD stages 3–5 according to antihyperglycemic drugs.

Antihyperglycemic Drugs	Total n (%)	CKD n (%)	No CKD n (%)	CKD Incident rate (95% CI) per 1000 person days	Log-rank test, *P*-value
**Sulfonylureas**					0.452
Yes	545 (54.3)	223 (40.9)	322 (59.1)	39.5 (34.7, 44.4)	
No	458 (45.7)	165 (36)	293 (64)	36.1 (30.8, 41.4)	
Thiazolidinedione					0.165
Yes	164 (16.4)	74 (45.1)	90 (54.9)	44.3 (34.8, 53.7)	
No	839 (83.6)	314 (37.4)	525 (62.6)	36.8 (33, 40.6)	
Dipeptidyl Peptidase					0.757
Yes	422 (42.1)	167 (39.6)	255 (60.4)	39 (33.5, 44.6)	
No	581 (57.9)	221 (38)	360 (62)	37.3 (32.6, 42)	
Biguanide					0.001
Yes	950 (94.7)	360 (37.9)	590 (62.1)	36.9 (33.2, 40.5)	
No	53 (5.3)	28 (52.8)	25 (47.2)	63.8 (42.3, 85.3)	
Alpha glucosidase					0.410
Yes	39 (3.9)	18 (46.2)	21 (53.8)	45.1 (25, 65.2)	
No	964 (96.1)	370 (38.4)	594 (61.6)	37.7 (34.1, 41.4)	
Insulin					< 0.001
Yes	227 (22.6)	123 (54.2)	104 (45.8)	57.2 (47.9, 66.6)	
No	776 (77.4)	265 (34.1)	511 (65.9)	33 (29.1, 36.7)	

**Abbreviations:** CKD; eGFR < 60 mL/min/1.73 m^2^ for ≥ 3 months. No CKD; eGFR > 60 mL/min/1.73 m^2^ for ≥ 3 months.

## Discussion

In our research, we have conducted a thorough investigation into the incidence of CKD at stages 3–5, analysed the impact of specific pharmaceutical interventions, and pinpointed significant predictive factors, thereby shedding light on the complex dynamics of CKD progression in individuals with type 2 diabetes. This discussion aims to interpret and contextualize findings within a broader context, providing deeper insights into the clinical implications, potential therapeutic strategies, and avenues for future research.

Our study included a cohort of 1,003 individuals. We observed 388 subjects developed CKD stages 3–5 across an average monitoring duration of 11.7 years. This resulted in a cumulative incidence of 38.7%, translating to an incidence rate of 38 cases per 1000 person-years. The data revealed a clear correlation between the use of statins and the progression of CKD. Individuals on high-intensity statin therapy were found to exhibit a greater propensity to progress to CKD stages 3–5 in comparison to with low/moderate intensity statin therapy or those not using statins at all, with incidence rates corroborating this finding. Conversely, the use of Biguanides was associated with a reduced likelihood of CKD progression, whereas Insulin users demonstrated a heightened risk.

The results of our investigation indicate a 38.7% incidence for progression to CKD stages 3 ± 5 over a mean follow-up period of 11.7 years, a figure notably higher than those reported in previous studies. These include Bash et al., 2008 (1.9%), Kim et al. (2014) (4.3%), Luk et al. (2013) (3%), Salinero-Fort et al. (2015) (2.2%), Shankar et al. (2007) (2.1%), Zoppini et al. (2012) (2.9%), and Yu et al. (2015) (23.8%). The observed discrepancy in the incidence of CKD stages 3–5 between our study and these previous investigations can potentially be attributed to variations in the baseline characteristics of respective study populations.

The incidence rate of CKD stages 3 ± 5 in our study was found to be 380.1 cases per 10,000 person-years, a figure unexpectedly higher than what was initially anticipated. In comparison, a Spanish prospective cohort study of 3,443 individuals with type 2 diabetes mellitus (DM) reported am incidence rate of 207 per 10,000 person-years for CKD stages 3–5 (Salinero-Fort et al., 2015). A separate study conducted in the United States on Pima Indians with type 2 DM showed incidence rates of 254 and 219 per 10,000 person-years for men and women, respectively (Vijayakumar et al., 2017). The elevated incidence rates observed in our current study may be attributable to a higher prevalence of patients presenting with cardiovascular disease (CVD) or those at an elevated risk for CVD. Interestingly, a comprehensive community-based cohort study conducted in Iran, which reported a lower prevalence of DM, still showed a higher incidence rate of CKD stages 3 ± 5, amounting to 214.82 per 10,000 person-years (Tohidi et al., 2012).

Statins are widely used to improve lipid profiles, and they have demonstrated efficacy in reducing albuminuria (Zhou et al., 2014), diabetic glomerulosclerosis in experimental animals (Vázquez-Pérez et al., 2001), and cardiovascular events (Jabir et al., 2016; Zhou et al., 2014). However, an ongoing debate persists regarding the effectiveness of statins in enhancing renal function in patients with diabetic nephropathy (Law & Rudnicka, 2006; Satirapoj et al., 2015). Additionally, the safety and benefits of high-intensity statin therapy in patients with chronic kidney disease (CKD) remain uncertain, a subject of significant interest among clinical professionals globally (Yan et al., 2015).

To the best of our knowledge, this is the first study to investigate the potential effects of statins on renal function in individuals with diabetic nephropathy in the United Arab Emirates.

Our study results indicate that individuals taking high-intensity statins have a higher incidence of developing CKD stages 3–5 compared to those not taking statins or receiving low to moderate doses. This finding aligns with another study indicating that high-potency statin therapy was associated with a 13% increased risk of severe renal failure compared to low-potency statins, a correlation observed across populations at risk, including those with diabetes, CKD, and ischemic heart disease (Chung et al., 2013). Furthermore, compared to patients receiving low-potency statin therapy, another study revealed a higher likelihood of hospitalisation for acute renal injury in those receiving high-potency statin therapy (Dormuth et al., 2013). Additionally, a meta-analysis found that high-dose rosuvastatin (40 mg/day) led to a higher incidence of new-onset proteinuria compared to low-dose rosuvastatin (Crouse et al., 2007).

Given that a significant portion of study participants had a history of cardiovascular illness or were at risk for it, this correlation between statin intensity and an increased prevalence of chronic renal disease can be explained. A Cochrane study further investigated our findings and found that statin medication consistently reduced mortality and prevented major cardiovascular events in individuals with CKD who did not require dialysis and did not have cardiovascular disease at baseline (Rodrigues, 2015).

Conversely, earlier research has suggested that statin medication may significantly slow the decline in the estimated glomerular filtration rate (eGFR) and possibly halt the progression of CKD (Palmer et al., 2014; Su et al., 2016). Similarly, a Canadian study observed that the benefits of statin therapy increased with dosage, with 37% of patients receiving high-potency statin therapy (Molnar et al., 2011). Another investigation revealed that high-dose atorvastatin (80 mg/day) improved renal outcomes compared to the low-dose group (10 mg/day) (Shepherd et al., 2007). Moreover, a meta-analysis of 10 randomised controlled trials (RCTs) found that statins significantly improved eGFR, with high-intensity statins yielding the most pronounced effects (Sanguankeo et al., 2015).

Nevertheless, an increasing body of research indicates that changes in estimated glomerular filtration rate did not significantly differ between the statin and control groups (Abe et al., 2011; Colhoun et al., 2009; Palmer et al., 2012; Satirapoj et al., 2015; Shen et al., 2016; Su et al., 2016; Zhao et al., 2021). Therefore, further high-quality research is required to examine the renal protective effects of high-intensity statins in patients with CKD, as there is ongoing disagreement regarding renal outcomes based on statin potency.

Our study results indicate that there was no statistically significant difference in the cumulative incidence of CKD stages 3–5 among different types of statins concerning renal function. In contrast to previous research, atorvastatin appears to offer more advantages than rosuvastatin (Crouse et al., 2007; de Zeeuw et al., 2015; Han et al., 2017). Furthermore, compared to the atorvastatin group in a prior randomised controlled trial, the rosuvastatin group demonstrated greater reductions in lipid profiles, significant GFR reductions, and a higher number of acute renal failure events (de Zeeuw et al., 2015). In a separate trial, the atorvastatin group experienced a smaller GFR decline compared to the rosuvastatin group (Crouse et al., 2007).

These findings hold significant implications for the treatment of individuals at risk of chronic kidney disease (CKD), particularly those with a history of hypertension, dyslipidemia, and ischemic heart disease. When prescribing statins for these individuals, healthcare professionals should carefully consider statin intensity, balancing the potential benefits of cholesterol management against the risk of developing CKD. High-intensity statin therapy should be used judiciously, taking into account each patient’s medical history and unique risk factors.

These results underscore the need for continued research into the relationship between renal function and statin therapy within the broader context of medical practice and research. Subsequent research efforts should validate these findings, explore underlying mechanisms driving the observed correlations, and refine protocols for statin administration in individuals at risk of chronic kidney disease.

This study contributes to the growing body of knowledge concerning the complexities of statin therapy in individuals at risk of CKD. It underscores the importance of personalised medicine, where treatment decisions are made with careful consideration of an individual’s specific health profile to optimise outcomes for both kidney and cardiovascular health.

The employment of metformin in the context of CKD has been a controversial topic, with its usage being substantially restricted in recent years due to concerns over potential associated cases of lactic acidosis (Calabrese et al., 2002; Kidney Disease: Improving Global Outcomes (KDIGO) Diabetes Work Group, 2020; Pugliese et al., 2020; Salpeter et al., 2010). Nevertheless, the results from our study suggest that individuals treated with Biguanides, a class of drugs that includes metformin, demonstrated a reduced likelihood for progressing to CKD stages 3-5. This observation aligns with contemporary findings related to CKD patients, highlighting a minimised risk of lactic acidosis and pointing to various beneficial effects that extend beyond the drug’s capacity to lower blood glucose levels. Notably, these effects include the potential to decelerate the deterioration of renal function (Charytan et al., 2019; Crowley et al., 2017; Ekström et al., 2012; Marcum et al., 2018; Roumie et al., 2019; Roussel et al., 2010; Zhang et al., 2020).

Furthermore, a recent retrospective observational study involving 10,862 type 2 diabetic patients with CKD provided promising results. Over an average monitoring period of 7.3 ± 4.8 years, individuals who were administered metformin exhibited a substantial reduction in mortality rates when compared with those who were not using metformin. The study also revealed a significant decrease in the progression to end-stage renal disease, as evidenced by the diminished need for renal replacement therapy. This advantageous impact on both mortality and the progression of nephropathy was most pronounced in patients who had eGFR levels ranging from 30 and 44 ml/min. Notably, these findings remained consistent even after the application of Propensity Score Matching and the utilisation of Kaplan–Meier curves for patient stratification based on KDIGO stage 3 and stage 4 (Kwon et al., 2020).

The findings of this study have established a link between the utilisation of insulin and an elevated risk of progression to CKD stages 3–5. The correlation is supported by a recent observational study, which highlighted a higher prevalence of insulin use in cases of more advanced CKD, and identified both insulin use and advanced CKD as independent risk factors for the occurrence of severe hypoglycemic events. Moreover, when compared to individuals with preserved kidney function who were not using insulin, the risk of serious hypoglycemic events was found to be approximately 5.3 times higher in patients who utilising insulin with an eGFR < 30 ml/min/1.73 m^2^ (Grube et al., 2022). This confirms prior findings from cross-sectional studies, which have also reported a higher incidence of insulin use among individuals with more advanced stages of CKD (Busch et al., 2016; Gor et al., 2019; Grandfils et al., 2013).

Despite offering insightful information about the effects of statin therapy and antihyperglycemic medications on the course of chronic kidney disease (CKD) in patients with Type 2 diabetic mellitus (T2DM), our study has a number of significant limitations. First, the sample size estimate suggested that 2580 participants would be needed to obtain the best statistical power; however, because of real-world limitations and data accessibility, our final cohort consisted of only 1,003 people. Due to the inability to achieve the required sample population, our findings may be less robust and generalizable. Additionally, although the *p*-value of 0.047 for the cumulative incidence of CKD stages 3–5 is statistically significant, the lower sample size implies that care should be taken when interpreting the results. Furthermore, selection bias may be introduced and the study’s generalizability to larger groups may be limited due to its retrospective design and dependence on electronic medical records (EMR) from a single location. The likelihood of residual confounding cannot be completely ruled out, even though we have corrected for potential confounders in our analysis.

Despite these limitations, our study has a number of noteworthy strengths that improve its overall integrity and quality. In order to gather a thorough range of CKD events, we implemented rigorous protocols, which included an 11.7-year follow-up period. To reduce the possibility of excluding possible cases of chronic kidney disease, our inclusion criteria were carefully crafted to include all eligible patients. We were able to gather comprehensive and current medical records because the strict selection criteria we applied ensured people missing baseline data or with less than a year of follow-up were not included. The timely reporting of CKD events was made easier by routine patient monitoring, which also decreased the likelihood of administrative censoring and follow-up loss. Every quarter, evaluations of each patient’s estimated glomerular filtration rate (eGFR) were carried out, enabling ongoing monitoring of their state. Comprehensive examinations of patient files were performed to identify and record any conflicting occurrences that could influence the course of CKD, making sure these were properly taken into account in our analysis.

## Conclusion

Our investigation sheds light on the influence of specific pharmaceutical interventions and treatment strategies on the progression of chronic kidney disease (CKD). Notably, we found a link between the use of statin therapy and the advancement of CKD stages 3–5. Particularly, individuals on high-intensity statin therapy were prone to progress in CKD stages compared to those on low/moderate intensity statins or those no using statins at all, with incidence rates confirming this pattern. This finding suggests the critical need for precision and individualised consideration in prescribing statins, especially for patients at higher risk of CKD. Furthermore, we examined the effects of other medications, such as Biguanides and Insulin, on the development of CKD. Results indicated that Biguanide users had a diminished risk of progressing to CKD stages 3–5, whereas Insulin users were found to be more susceptible to its onset. These results emphasise the importance of tailoring medication regimens to the unique clinical profiles and medical histories of individual patients.
